# Differential transcriptomic profiles effected by oil palm phenolics indicate novel health outcomes

**DOI:** 10.1186/1471-2164-12-432

**Published:** 2011-08-25

**Authors:** Soon-Sen Leow, Shamala Devi Sekaran, Kalyana Sundram, YewAi Tan, Ravigadevi Sambanthamurthi

**Affiliations:** 1 Malaysian Palm Oil Board, No. 6, Persiaran Institusi, Bandar Baru Bangi, 43000 Kajang, Selangor, Malaysia; 2 University of Malaya, 50603 Kuala Lumpur, Malaysia; 3 Malaysian Palm Oil Council, 2nd Floor, Wisma Sawit, Lot 6, SS6, Jalan Perbandaran, 47301 Kelana Jaya, Selangor, Malaysia

## Abstract

**Background:**

Plant phenolics are important nutritional antioxidants which could aid in overcoming chronic diseases such as cardiovascular disease and cancer, two leading causes of death in the world. The oil palm (*Elaeis guineensis*) is a rich source of water-soluble phenolics which have high antioxidant activities. This study aimed to identify the *in vivo *effects and molecular mechanisms involved in the biological activities of oil palm phenolics (OPP) during healthy states via microarray gene expression profiling, using mice supplemented with a normal diet as biological models.

**Results:**

Having confirmed via histology, haematology and clinical biochemistry analyses that OPP is not toxic to mice, we further explored the gene expression changes caused by OPP through statistical and functional analyses using Illumina microarrays. OPP showed numerous biological activities in three major organs of mice, the liver, spleen and heart. In livers of mice given OPP, four lipid catabolism genes were up-regulated while five cholesterol biosynthesis genes were down-regulated, suggesting that OPP may play a role in reducing cardiovascular disease. OPP also up-regulated eighteen blood coagulation genes in spleens of mice. OPP elicited gene expression changes similar to the effects of caloric restriction in the hearts of mice supplemented with OPP. Microarray gene expression fold changes for six target genes in the three major organs tested were validated with real-time quantitative reverse transcription-polymerase chain reaction (qRT-PCR), and the correlation of fold changes obtained with these two techniques was high (R^2 ^= 0.9653).

**Conclusions:**

OPP showed non-toxicity and various pleiotropic effects in mice. This study implies the potential application of OPP as a valuable source of wellness nutraceuticals, and further suggests the molecular mechanisms as to how dietary phenolics work *in vivo*.

## Background

The continuous improvement in the quality of life in recent years, together with the advancement of science and technology, has created a more health-conscious society. Data from large population studies suggest that lifestyle factors, such as sedentary lifestyle, dietary intake and adiposity are responsible for 70% of chronic diseases and are a major contributor to reduced longevity [1]. Thus, society now realises the importance of physical activity and dietary intervention towards the prevention of chronic diseases and ageing. Chronic diseases such as cardiovascular disease and cancer are by far the leading cause of mortality in the world, representing 60% of all deaths [2].

Prooxidants such as reactive oxygen species (ROS) play important roles in triggering chronic diseases. For example, the hydroxyl radical causes lipid peroxidation, modification of DNA bases or protein damage and in turn, leads to tissue damage, chronic diseases and ageing [3]. In addition to oxidising macromolecules such as DNA in the body, prooxidants have been found to regulate gene expression, of which two well-defined transcription factors, nuclear factor kappa-B and activator protein-1 are affected, causing inflammation [4]. Inflammation thus links oxidative stress with chronic diseases.

Many studies conducted recently have shown that diets containing high amounts of phytochemicals can provide protection against these prooxidant-induced diseases, due to their high antioxidant activities. Antioxidants preclude prooxidant-induced tissue damage by preventing the formation of these prooxidants, by scavenging them or by promoting their decomposition [3]. Antioxidants are also found to be caloric restriction mimetics, the only intervention known to lengthen the median lifespan of animals [5]. Among the different groups of naturally occurring antioxidants from plants, carotenoids and phenolics are perhaps the two most important [6]. Phenolics comprise an aromatic ring, and are important antioxidants because of their high redox potential, which allows them to act as reducing agents, hydrogen donors, singlet oxygen quenchers and metal chelators [6]. Around 8000 phenolic compounds have been identified thus far, and they have been classified into different categories based on their basic structures [7]. Phenolics possess many therapeutic properties. For example, flavonoids have been shown to have antioxidant, neuroprotective, cardioprotective, lipid-lowering, anti-hepatotoxic, anti-allergic, anti-cancer, anti-inflammatory, anti-thrombotic and anti-microbial activities [8].

Besides their antioxidant properties, phytochemicals such as phenolics are also known to influence gene expression [9]. Microarray analysis has thus been suggested as a tool to identify modulations of multiple gene networks by antioxidant micronutrients [10], as the expression profiles of thousands of genes can be measured in a single experiment [11]. As dietary intervention generally results in small effects on a big number of genes compared to pharmaceutical intervention which targets a specific biomarker, microarrays can therefore detect a combined effect of several genes belonging to a similar biological pathway [12]. Indeed, multi-component botanical therapeutics such as phenolics may become particularly valuable in the long-term prevention and treatment of complex diseases requiring extended administration and pleiotropic action [13]. In studies on plant phenolics, microarray studies have been carried out to test the biological effects of flavonoids in cultured cell lines *in vitro *[14,15] as well as in animals *in vivo *[12].

The oil palm (*Elaeis guineensis*) contains an excellent repertoire of antioxidant phytochemicals. Palm fruit oil contains many lipid-soluble antioxidants, such as carotenoids (precursors of vitamin A), as well as tocopherols and tocotrienols (isomers of vitamin E) [16-18]. Another relatively new antioxidant phytochemical from the oil palm fruit is the water-soluble phenolic acid-rich complex, which is recovered from the oil palm vegetation liquor through a series of centrifugation and membrane filtration steps [19]. These oil palm phenolics (OPP) consist mainly of phenolic acids, including three caffeoylshikimic acid isomers, protocatechuic acid and *p*-hydroxybenzoic acid [20,21]. OPP has been shown to display antioxidant properties and confer positive outcomes on degenerative diseases in various animal models without evidence of causing toxicity [21-23]. The utilisation of OPP in the nutraceutical market is however, subject to understanding the molecular mechanisms involved in bringing about the positive benefits observed, whether in healthy or diseased states. The aim of this study was thus to identify the *in vivo *biological effects and molecular mechanisms of these compounds during healthy states in three major organs (liver, spleen and heart) of mice on a normal diet, via microarray gene expression profiling.

## Results

### Weight gain delay and non-toxicity in mice supplemented with OPP

**Figure 1:**
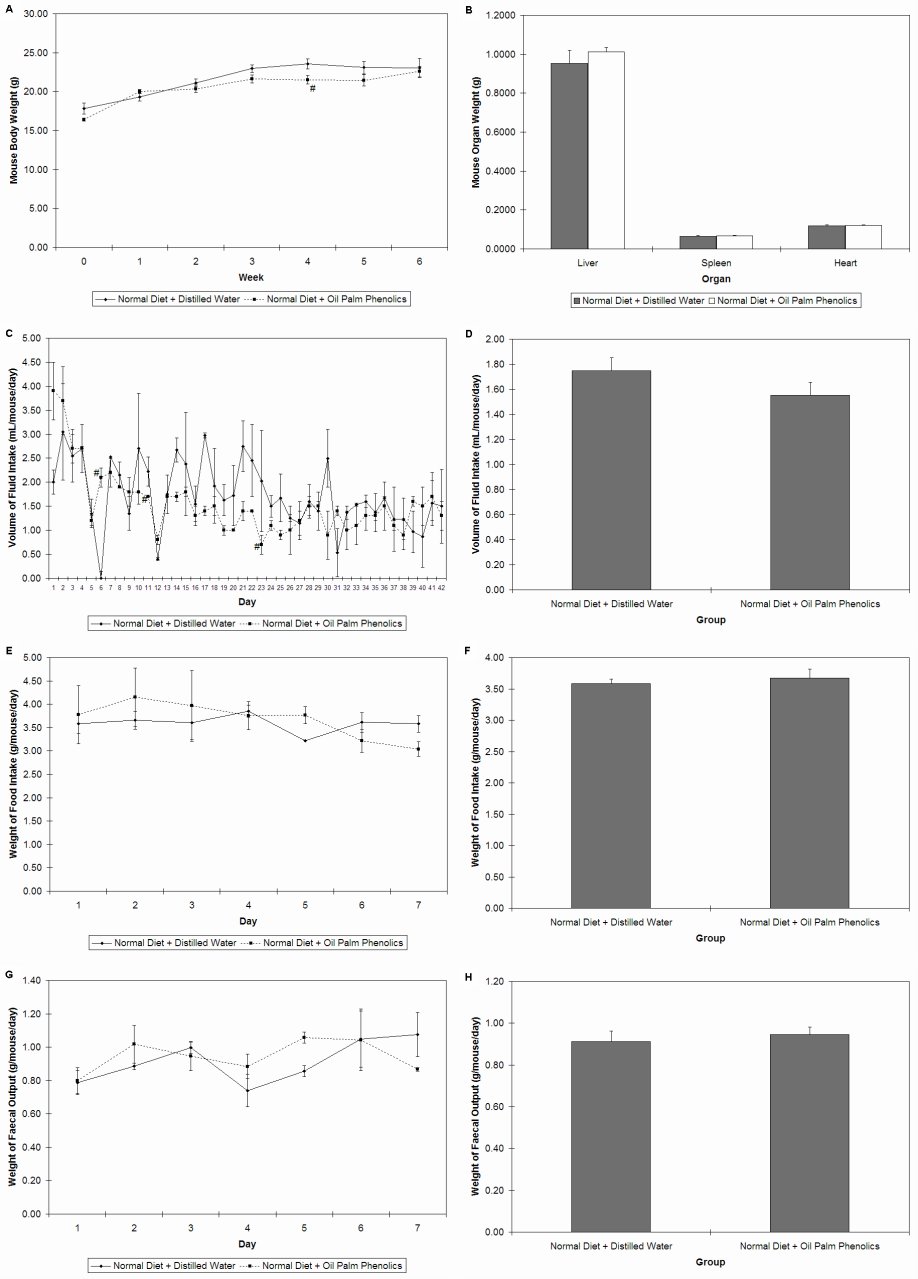
**Physiology parameters of mice**. **(A) **Body weights; n = 10. **(B) **Organ weights; n = 10. **(C) **Timeline of fluid intake; n = 2 cages (of 5 mice per cage). **(D) **Average daily fluid intake; n = 42 days. **(E) **Timeline of food intake, n = 2 cages (of 5 mice per cage). **(F) **Average daily food intake between week two to week three; n = 7 days. **(G) **Timeline of faecal output, n = 2 cages (of 5 mice per cage). **(H) **Average daily faecal output between week two to week three; n = 7 days. # P < 0.05 vs. Normal Diet + Distilled Water. Error bars indicate s.e.m.

**Figure 2:**
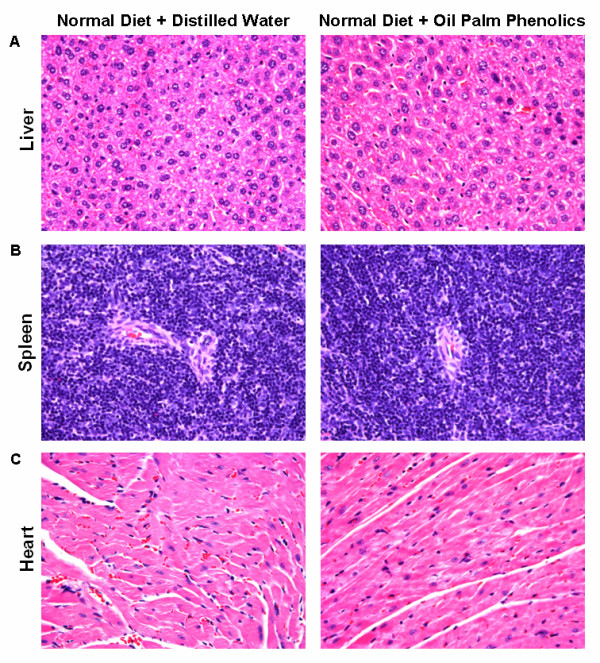
**OPP did not affect organ histology**. Representative haematoxylin and eosin stained tissue slices from the three major organs in each group were viewed under a light microscope with a magnification of X200. **(A) **Liver. **(B) **Spleen. **(C) **Heart.

We first confirmed that OPP was not toxic to mice via physiology, histology, haematology and clinical biochemistry analyses. The body weights of mice steadily increased every week throughout the six weeks of feeding, with mice given OPP showing a delayed weight gain (Figure 1A). OPP did not significantly affect organ weights (Figure 1B), fluid intake (Figures 1C and 1D), food intake (Figures 1E and 1F), faecal output (Figures 1G and 1H), organ histology (Figure 2), as well as haematology and clinical biochemistry (Table 1) parameters.

**Table 1:** Haematology and clinical biochemistry parameters of mouse blood samples

Test	Normal Diet + Distilled Water	Normal Diet + Oil Palm Phenolics
Haematology	(*n = 4*)	(*n = 4*)
Red Blood Cells (X10^12^/L)	9.93 ± 0.32	10.15 ± 0.12
Haemoglobin (g/L)	148 ± 4	149 ± 1
Haematocrit/Packed Cell Volume (L/L)	0.40 ± 0.01	0.40 ± 0.00
Mean Corpuscular Volume (fL)	41 ± 1	40 ± 0
Mean Corpuscular Haemoglobin Concentration (g/L)	369 ± 6	373 ± 4
White Blood Cells (X10^9^/L)	2.0 ± 0.6	1.5 ± 0.3
Band Neutrophils (X10^9^/L)	0.05 ± 0.01	0.04 ± 0.01
Segmented Neutrophils (X10^9^/L)	0.48 ± 0.17	0.35 ± 0.09
Lymphocytes (X10^9^/L)	1.36 ± 0.38	1.01 ± 0.20
Monocytes (X10^9^/L)	0.09 ± 0.02	0.07 ± 0.02
Eosinophils (X10^9^/L)	0.03 ± 0.01	0.01 ± 0.00
Basophils (X10^9^/L)	0.00 ± 0.00	0.00 ± 0.00
Thrombocytes (X10^9^/L)	533 ± 111	621 ± 103
Prothrombin (g/L)	79 ± 2	80 ± 1
Clinical Biochemistry	(*n = 8*)	(*n = 7*)
Alanine Aminotransferase (U/L)	34.4 ± 3.3	42.5 ± 6.5
Aspartate Aminotransferase (U/L)	175.2 ± 23.8	240.4 ± 22.3
Glucose (mmol/L)	6.0 ± 1.1	6.7 ± 0.4
Serum Total Protein (g/L)	53.8 ± 1.8	53.8 ± 1.1
Albumin (g/L)	34.0 ± 0.9	33.1 ± 1.3
Globulin (g/L)	19.8 ± 1.1	20.8 ± 0.8
Albumin:Globulin	1.8 ± 0.1	1.6 ± 0.1
Total Cholesterol (mmol/L)	3.46 ± 0.13	3.53 ± 0.19
Triglycerides (mmol/L)	1.05 ± 0.08	1.04 ± 0.11
Low-Density Lipoproteins (mmol/L)	0.15 ± 0.02	0.18 ± 0.03
High-Density Lipoproteins (mmol/L)	2.79 ± 0.11	2.83 ± 0.17

Values shown are means ± s.e.m. Statistical analysis did not show significant differences in all the parameters measured.

### Overview of gene expression changes in the liver, spleen and heart

Illumina microarrays were used to study the effects of OPP in the livers, spleens and hearts of mice given a normal diet. OPP significantly changed 250 genes in the liver (196 genes up-regulated and 54 genes down-regulated), 142 genes in the spleen (100 genes up-regulated and 42 genes down-regulated), and 475 genes in the heart (196 genes up-regulated and 279 genes down-regulated). The number of genes significantly changed by OPP was thus highest in the heart, followed by the liver and the spleen. The lists of genes significantly changed by OPP in these organs, together with their fold changes, are supplemented in Additional File 1.

The effects of OPP on the organs tested were quite subtle. The majority of the differentially expressed genes rarely exceeded two fold change in expression, with the exception of genes considered turned on (significantly detected only in the treatment group) or off (significantly detected only in the control group). The large fold changes observed in this particular group of genes (turned on or off) are arbitrary values as values of 10 were assigned when negative expression values were observed after normalisation. These results indicate that OPP can act as regulators of gene expression, turning on and silencing genes. In addition, the low fold changes caused by OPP indicate that they do not have drastic effects on gene expression in general, and may be used as dietary supplements. Such supplements should not bring too drastic a change in the system of an organism, but rather act as an antioxidant buffer to cushion the effects of other environmental influences such as prooxidant production.

An example of the two-way hierarchical clustering analysis carried out on significantly changed genes in the liver is given in Additional File 2. The replicates of each condition were clustered to one another, indicating robustness of the filtering criteria used. In order to get an overview of the significant functions affected by OPP, we then subjected the microarray data to functional enrichment analysis using the GenMAPP [24] and MAPPFinder [25] softwares (University of California at San Francisco, San Francisco, CA). The lists of these significantly changed functions are provided in Additional File 3. By exploring functions which were considered significantly changed, we further selected GenMAPPs which were considered interesting for pathway analysis. Network analysis was also carried out on the differentially expressed genes by using the Ingenuity Pathways Analysis software (Ingenuity^® ^Systems, Redwood City, CA) [26].

### Gene expression changes in the liver

**Figure 3:**
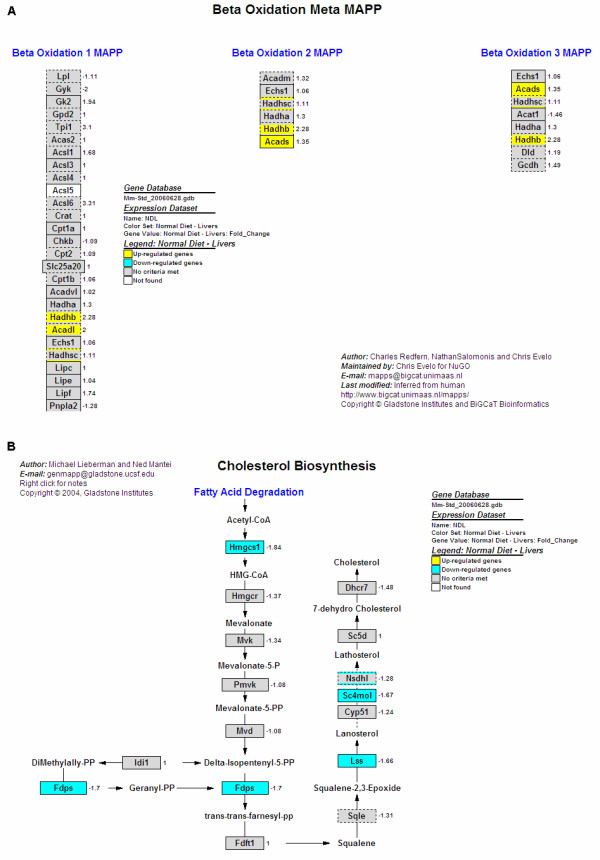
**GenMAPPs showing functions and genes significantly changed by OPP in the liver**. **(A) **Genes up-regulated in the liver fatty acid beta oxidation pathway. This GenMAPP represents an overview of three individual fatty acid beta oxidation GenMAPPs, and shows the up-regulation of fatty acid beta oxidation genes such as acetyl-CoA dehydrogenase (*Acadl*), acyl-CoA dehydrogenase (*Acads*) and hydroxyacyl-CoA dehydrogenases (*Hadhb, Hadhsc*). **(B) **Genes down-regulated in the liver cholesterol biosynthesis pathway. Note that the fold changes for most of the genes in this GenMAPP were negative, indicating down-regulation, even for genes which were not selected as significantly different based on the selection criteria used. Also note that *Hmgcr *which encodes for 3-hydroxy-3-methylglutaryl-CoA reductase, an enzyme inhibited by cholesterol-lowering statins, showed a negative fold change as well, although the value was not statistically significant.

In the GenMAPPs and gene ontologies found significantly changed in the liver, those related to fatty acid beta oxidation (for up-regulated genes) and cholesterol biosynthesis (for down-regulated genes), were predominant. Further analysis on the fatty acid beta oxidation GenMAPP (pathway) revealed the up-regulation of genes such as those encoding long chain acetyl-CoA dehydrogenase (*Acadl*), short chain acyl-CoA dehydrogenase (*Acads*) and hydroxyacyl-CoA dehydrogenases (*Hadhb, Hadhsc*) (Figure 3A). These significantly up-regulated genes are highlighted in yellow.

Other functions which were significantly up-regulated by OPP in the liver were complement activation, protein biosynthesis and transport, ion and electron transport as well as translation factors. Up-regulated genes involved in complement activation include *C1r, C1rl, C1s, C4, C9 *and *Cfhl1*. In addition, genes involved in ion and electron transport such as those encoding cytochromes P450 involved in phase I metabolism (*Cyp1a2, Cyp27a1, Cyp2a12, Cyp2c54, Cyp2d26, Cyp3a11*) and NADH dehydrogenase (ubiquinone) Fe-S proteins (*Ndufs2, Ndufs3*), were up-regulated. Genes involved in phase II metabolism such as those encoding UDP glycosyltransferases (*Ugt1a6 *and *Ugt1a9*) and catechol-O-methyltransferase (*Comt*), were up-regulated as well. Translation factors involved in protein synthesis (*Eef1d, Eef2, Eif3s2, Eif3s8, Eif4a2, Eif5a*) were also up-regulated by OPP.

Genes involved in cholesterol biosynthesis on the other hand, such as those encoding lanosterol synthase (*Lss*), sterol-C4-methyl oxidase-like (*Sc4mol*), farnesyl diphosphate synthetase (*Fdps*), NAD(P) dependent steroid dehydrogenase-like (*Nsdhl*) and 3-hydroxy-3-methylglutaryl-CoA synthase 1 (*Hmgcs1*) were down-regulated (Figure 3B). In this pathway, besides those highlighted in blue (genes which were significantly down-regulated), other genes in the pathway had negative fold changes, the most important being *Hmgcr *(hydroxymethylglutaryl-CoA reductase), a gene targeted by drugs such as statins for lowering cholesterol. The fold change difference of this gene was however, not significant due to the filtering criteria used.

### Gene expression changes in the spleen

**Figure 4:**
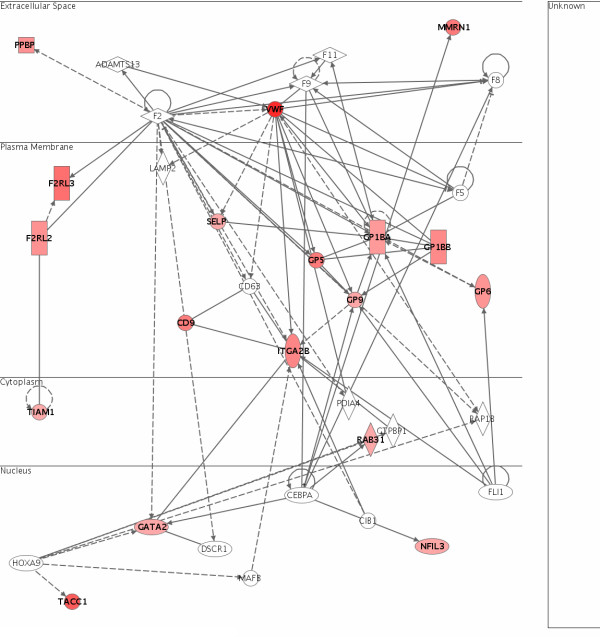
**Genes involved in the spleen blood coagulation network which were up-regulated by OPP**. The up-regulation of various genes involved in blood coagulation such as those encoding Von Willebrand factor homologue (*Vwf*), P-selectin (*Selp*), various glycoproteins (*Gp1ba, Gp1bb, Gp5, Gp6, Gp9*) and thrombin receptors (*F2rl2 *and *F2rl3*), suggests a possible effect of OPP in clearing blood clots from the circulation via the spleen.

A majority of the genes significantly up-regulated in the spleen were part of the blood coagulation network (Figure 4). Some of the up-regulated genes were those encoding Von Willebrand factor homologue (*Vwf*), P-selectin (*Selp*), CD9 antigen (*Cd9*), integrin alpha 2b (*Itga2b*), multimerin 1 (*Mmrn1*), various glycoproteins (*Gp1ba, Gp1bb, Gp5, Gp6, Gp9*) and thrombin receptors (*F2rl2, F2rl3*). These genes are indicated in red in the blood coagulation network.

While a pathway involves the metabolism of biochemical compounds catalysed by enzymes, a network indicates the relationship of one protein to another. A network analysis may thus help to detect other possible proteins affected by the treatment. For example, a number of coagulation factors (*F2, F5, F8, F9, F11*) was found to be connected to the genes significantly up-regulated in the spleen blood coagulation network. These genes might thus be up-regulated and contribute to the blood coagulation process observed in this experiment as well, although the statistical threshold used might not result in these genes being selected as significantly changed. A network analysis can also help in the identification of central regulators in specific networks. For example, the *Vwf *gene appeared to be the central regulator in the blood coagulation network, and might thus play a central role in this process by up-regulating the other genes in response to OPP treatment.

In addition to blood coagulation, other functions significantly up-regulated in the spleen by OPP include actin binding, calcium ion binding, cell adhesion, fatty acid metabolism, focal adhesion, prostaglandin synthesis regulation, sugar binding and tumour necrosis factor binding. Genes down-regulated in the spleen on the other hand, were involved in DNA replication, interleukin-9 signalling, nitrogen metabolism and unfolded protein binding.

### Gene expression changes in the heart

**Figure 5:**
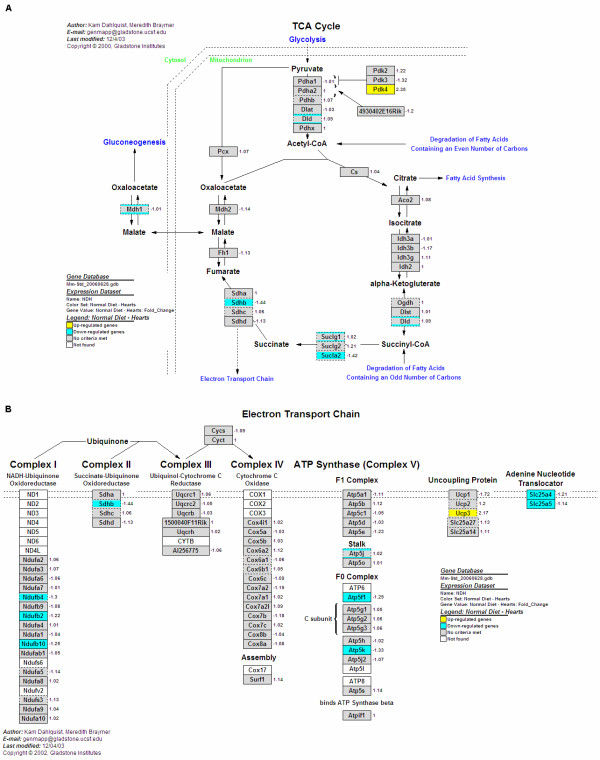
**GenMAPPs showing functions and genes significantly changed by OPP in the heart**. **(A) **Genes up-regulated and down-regulated in the heart tricarboxylic acid (TCA) cycle. *Pdk4*, which is involved in fuel selection in the heart by inhibiting pyruvate dehydrogenase and preventing the metabolic shift in ageing hearts from fatty acid beta oxidation towards glycolysis, was up-regulated, while other genes such as *Mdh1, Shdb, Suclg1, Sucla2 *and *Dld *were down-regulated. **(B) ***Ucp3*, a gene encoding an uncoupling protein which protects against mitochondrial oxidative damage by reducing the production of reactive oxygen species (ROS), was up-regulated, while other genes which encode adenine nucleotide translocators and proteins in Complex I, Complex II and Complex V in the electron transport chain were down-regulated.

In the heart, genes up-regulated by OPP were involved in acetyl-CoA, butanoate, fatty acid and lipid metabolisms, golgi organisation and biogenesis, glutathione metabolism, magnesium ion binding, nitric oxide mediated signal transduction and translation factors. Down-regulated genes in the heart include those involved in energy production, such as the tricarboxylic acid (TCA) cycle (Figure 5A) and the electron transport chain (Figure 5B). Genes involved in protein biosynthesis such as chromatin assembly or disassembly, haem biosynthesis, mitochondrial genome maintenance, mRNA processing and binding, as well as those encoding ribosomal proteins, were also down-regulated.

In the heart TCA cycle, a key enzyme, pyruvate dehydrogenase kinase isoenzyme 4 (*Pdk4*), which inhibits pyruvate dehydrogenase and thus minimises carbohydrate oxidation by preventing the flow of glycolytic products into the TCA cycle, was up-regulated. In conjunction with this, the expression of other genes involved in the heart TCA cycle, such as those which produce NADH (*Mdh1*) and FADH_2 _(*Shdb*), were down-regulated as well. *Suclg1 *and *Sucla2*, genes encoding subunits of succinate-CoA ligases which convert guanosine triphosphate/adenosine triphosphate (GTP/ATP) to guanosine diphosphate/adenosine diphosphate (GDP/ADP) were also down-regulated.

In the heart electron transport chain, *Ucp3*, a gene encoding an uncoupling protein which protects against mitochondrial oxidative damage by reducing the production of ROS, was up-regulated as well. On the other hand, genes in the electron transport chain which encode proteins in Complex I *(Ndufb2, Ndufb4, Ndufb10, Ndufc2, Ndufs4*), Complex II (*Sdhb*) as well as those in Complex V (*Atp5fi, Atp5j, Atp5k, Atp6v0c, Atp6v1d*), were down-regulated.

### Real-time qRT-PCR validation

**Figure 6:**
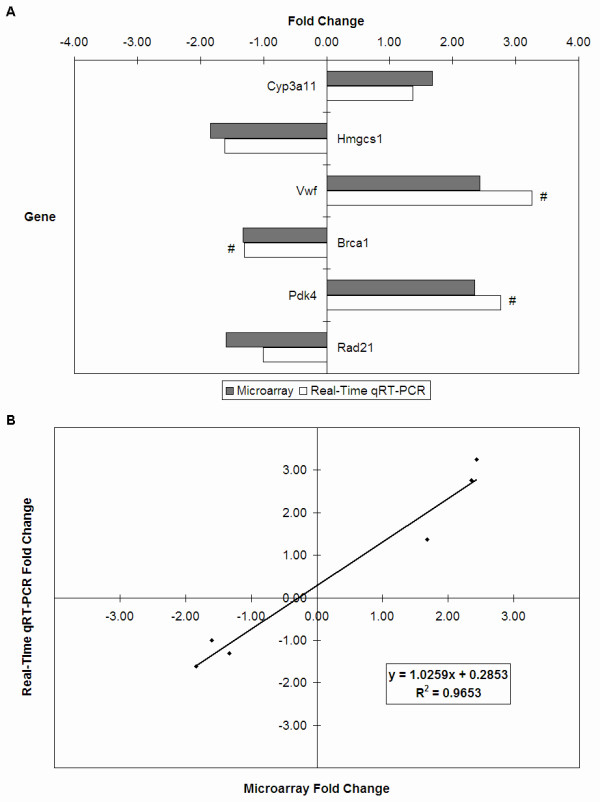
**Real-time qRT-PCR validation of microarray data analysis on the three major organs**. **(A) **Gene expression fold changes of six target genes as determined by microarray and real-time qRT-PCR experiments. The direction and magnitude of fold changes obtained from the real-time qRT-PCR technique were comparable to those obtained from the microarray technique. # P < 0.05 for gene expression fold changes quantified by real-time PCR experiments as determined by two-tailed unpaired Student's t-test. **(B) **Correlation of gene expression fold changes between microarray and real-time qRT-PCR data. Validation of the microarray data via real-time qRT-PCR shows that correlation of fold changes obtained by these two gene expression profiling techniques was high with an R^2 ^= 0.9653.

To confirm the microarray results, the expression levels of six target genes (Table 2) were measured using real-time quantitative reverse transcription-polymerase chain reaction (qRT-PCR). Expression levels of these target genes were normalised to the geometric mean of three housekeeping genes, *Sfrs9, Guk1 *and *Hnrpab*. The direction and magnitude of fold changes of the target genes obtained from the real-time qRT-PCR technique were comparable to those obtained from the microarray technique (Figure 6A). Correlation of fold changes obtained by the two gene expression profiling techniques was high (R^2 ^= 0.9653) (Figure 6B), thus indicating that the microarray data obtained were valid.

**Table 2:** Genes selected for the real-time qRT-PCR validation experiments

Organ	Symbol	Definition	Accession	Assay ID
Liver	*Cyp3a11*	*Mus musculus *cytochrome P450, family 3, subfamily a, polypeptide 11	NM_007818	Mm00731567_m1
Liver	*Hmgcs1*	*Mus musculus *3-hydroxy-3-methylglutaryl-Coenzyme A synthase 1	NM_145942	Mm00524111_m1
Spleen	*Vwf*	*Mus musculus *Von Willebrand factor homologue	NM_011708	Mm00550376_m1
Spleen	*Brca1*	*Mus musculus *breast cancer 1	NM_009764	Mm00515386_m1
Heart	*Pdk4*	*Mus musculus *pyruvate dehydrogenase kinase, isoenzyme 4	NM_013743	Mm00443325_m1
Heart	*Rad21*	*Mus musculus *RAD21 homologue (*S. pombe*)	NM_009009	Mm00485474_m1
All	*Sfrs9*	*Mus musculus *splicing factor, arginine/serine rich 9	NM_025573	Mm00470546_m1
All	*Guk1*	*Mus musculus *guanylate kinase 1	NM_008193	Mm00433888_m1
All	*Hnrpab*	*Mus musculus *heterogeneous nuclear ribonucleoprotein A/B	NM_010448	Mm00468938_m1

These six target genes were selected based on their presence in significant functions, their differential scores, their detection levels, their presence as single splice transcripts in microarrays and their availability as Taqman assays designed across splice junctions. *Sfrs9, Guk1 *and *Hnrpab *were used as housekeeping genes as their expression levels were found to be quite stable across treatments in each of the organs tested.

## Discussion

As OPP displayed significant antioxidant activities and conferred positive outcomes in various animal models of degenerative diseases without causing toxicity [21-23], we hypothesised that it might also have significant biological effects in animals during healthy states. Thus, we fed BALB/c mice a normal diet with OPP for six weeks and looked for signs of toxicity to confirm that OPP is safe. Microarray gene expression analysis was then carried out on three major organs, the liver, spleen and heart. This study thus represents an important step towards understanding the mode of action of OPP during healthy states, before further studies are carried out to identify the molecular mechanisms involved during diseased states.

### OPP up-regulated fatty acid beta oxidation genes and down-regulated cholesterol biosynthesis genes in the liver

In identifying the *in vivo *gene expression changes caused by any dietary intervention via microarray analysis, the liver naturally becomes the target organ of interest [27,28]. This is because it is a vital organ for various metabolic functions, including detoxification and production of biochemicals necessary for digestion and maintenance of body processes. In this study, functional enrichment analysis on the microarray data from the liver showed that OPP up-regulated several pathways, including lipid catabolism (fatty acid beta oxidation), complement activation, protein biosynthesis and transport, ion and electron transport as well as translation factors.

As the liver is an important site for fatty acid beta oxidation, up-regulation of hepatic lipid catabolism may contribute to the suppression of liver fat and visceral fat accumulation. Stocker and Keaney (2004) suggested that removal of lipids from the body through fatty acid beta oxidation may prevent lipid peroxidation which contributes to atherosclerosis [29]. Interestingly, enhanced hepatic fatty acid synthesis and reduced fatty acid oxidation have also been implied in the development of alcohol-induced fatty liver [30-33]. Thus, OPP may also be able to reduce atherosclerosis and prevent alcohol-induced liver damage based on its ability to up-regulate hepatic fatty acid beta oxidation. Increased expression of hepatic genes involved in lipid catabolism has also been shown to be effected by the catechins of green tea [34] and the chlorogenic acids of coffee [35].

The up-regulation of genes involved in complement activation observed in the present study, is similar to that reported for resveratrol [36]. In the resveratrol study, complement pathways, both classical and alternative, as well as acute inflammatory response, were up-regulated for reasons unknown, although the authors noted that no evidence of widespread inflammatory response was observed. The induction of the classical complement pathway by OPP may be triggered by antibodies, as plant antioxidants were reported to enhance immunity [37]. As an important component of innate immunity, complement activation is necessary for protection against numerous microbial infections [38,39].

Genes involved in ion and electron transport such as those encoding cytochromes P450 and NADH dehydrogenase (ubiquinone) Fe-S proteins were also up-regulated by OPP. In general, phenolics may be regarded as xenobiotics by animal cells and are to some extent treated as such through interaction with phase I and phase II detoxification enzyme systems [40]. Phase I detoxification in the liver involves the activation of a series of enzymes called the cytochrome P450 mixed-function oxidases. These enzymes begin the biotransformation process by oxidising, reducing or hydrolysing toxins, thus creating biotransformed intermediates [41]. A phase I detoxification gene of special interest is *Cyp3a4*, which encodes the most abundant cytochrome P450 in the human liver (around 30%), responsible for 60% of cytochrome P450-mediated metabolism of drugs in therapeutic use [42]. *Cyp3a4 *was found to be induced more than 2-fold by dietary flavonoids such as quercetin and grape seed extract in HepG2 hepatocytes [42]. *Cyp3a11 *is the mouse homologue of the human *Cyp3a4 *[43]. It is interesting to note that OPP up-regulated *Cyp3a11 *1.68-fold in the mouse liver. Incidentally, *Cyp3a11 *was up-regulated in the mouse liver as well by α-tocopherol [44]. This indicates that OPP has similar effects to other dietary antioxidants and may thus be involved in drug-herbal/botanical interaction effects. Phase II detoxification enzymes such as UDP glycosyltransferases perform conjugation reactions which help to convert biotransformed intermediates into less toxic, water-soluble substances that are easily excreted or eliminated from the body [41]. In the present study, genes *Ugt1a6 *and *Ugt1a9 *encoding UDP glycosyltransferases were up-regulated 2.11-fold and 1.95-fold respectively. *Comt*, the gene which encodes catechol-O-methyltransferase, an enzyme involved in tyrosine metabolism, was also up-regulated by OPP. Catechol-O-methyltransferase has been found to be involved in the metabolism of hydroxytyrosol, the major component of olive phenolics [45]. The up-regulated translation factors involved in protein synthesis, may also reflect the production of extra enzymes which help in metabolising OPP.

Other genes up-regulated by OPP are those encoding chemokine (C-X-C motif) ligand 12 (*Cxcl12*), gap junction membrane channel protein beta 1 (*Gjb1*) and androgen receptor (*Ar*), which were 1.87-, 1.38- and 2.30-fold increased respectively. *Cxcl12 *is a gene encoding a chemokine modulating the progression of liver fibrosis through its action on hepatic stellate cells [46], while *Gjb1 *encodes a gap junction membrane channel protein which plays an important role in the regulation of signal transfer and growth control in the liver. The expression of these genes decreased as liver diseases progress to cirrhosis and hepatocellular carcinoma [47]. Both of these genes were down-regulated by comfrey (*Cxcl12 *down-regulated 5.26-fold, *Gjb1 *down-regulated 2.08-fold), a herbal plant containing pyrrolizidine alkaloids believed to cause hepatotoxicity in humans and carcinogenicity in animals [46]. *Ar *was also down-regulated by comfrey (13.6-fold), as well as by riddelliine (19.6-fold) which also contains pyrrolizidine alkaloids [48]. Hence, the up-regulation of these three genes indicates that OPP may have hepatoprotective effects, and also implies that they do not cause hepatotoxicity.

Genes involved in cholesterol biosynthesis on the other hand, were down-regulated by OPP in this study. Cholesterol is an important constituent of cellular membranes and serves as a precursor in the formation of bile acids and steroid hormones. Excessive cholesterol however, is implicated in atherosclerotic lesions and gallstone formation [49]. The reduced expression of genes involved in cholesterol biosynthesis in the mouse liver suggests a possible role of OPP in reducing atherosclerosis and hence, cardiovascular disease. Overall, the gene expression changes caused by OPP in the liver suggest hepatoprotective and anti-dyslipidaemic effects of the extract.

### OPP up-regulated blood coagulation genes in the spleen

In the spleen, the most interesting genes up-regulated by OPP were those involved in blood coagulation. The regulation of these genes suggests that the spleen might be playing an active protective role in improving blood circulation and clearing blood clots from the body. OPP may thus function as anti-thrombotic agents, considering the fact that as a peripheral lymphoid organ, the spleen is also involved in mechanical filtration of the blood to remove unwanted materials [50]. However, this is still hypothetical and further experiments are required to show that OPP helps in clearing and not causing blood clots.

### Gene regulation by OPP in the heart showed similarities with that of caloric restriction

Ageing in the heart involves a transcriptional shift from fatty acid metabolism to carbohydrate metabolism. This shift was also found to differentiate maladaptive hypertrophy (increased glycolysis) from adaptive or exercise-induced hypertrophy (increased fatty acid beta oxidation) [51]. A key enzyme, pyruvate dehydrogenase kinase isoenzyme 4 (*Pdk4*), inhibits pyruvate dehydrogenase and thus minimises carbohydrate oxidation by preventing the flow of glycolytic products into the TCA cycle. Hence, the up-regulation of *Pdk4 *in the heart by OPP may prevent a metabolic shift towards glycolysis and thus ageing. Another gene, *Ucp3*, encoding an uncoupling protein which protects against mitochondrial oxidative damage by reducing the production of ROS, was up-regulated in the heart by OPP as well. *Ucp3 *may transport fatty acids out of the mitochondria, thereby protecting the mitochondria from fatty acid anions or peroxides [52]. Besides up-regulating *Pdk4 *and *Ucp3*, OPP also up-regulated another seven genes in the heart, carnitine acetyltransferase (*Crat*), catechol-O-methyltransferase (*Comt*), CCAAT/enhancer binding protein (*Cebpb*), enoyl-CoA, hydratase/3-hydroxyacyl CoA dehydrogenase (*Ehhadh*), plasma membrane associated protein (*S3-12*), solute carrier family 27 (fatty acid transporter) (*Slc27a1*) and xanthine dehydrogenase (*Xdh*). Lee *et al*. (2002) demonstrated the down-regulation of these nine genes in the hearts of ageing B6C3F_1 _mice [53]. Six of these genes, *Pdk4, Ucp3, Comt, Cebpb, S3-12 *and *Xdh *were significantly up-regulated by caloric restriction, thus opposing the effects of ageing [53]. Additional genes up-regulated by OPP and also by caloric restriction in the heart include carbonic anhydrase 4 (*Car4*), carboxylesterase 3 (*Ces3*), purine-nucleoside phosphorylase (*Pnp*) and tumour protein D52-like 1 (*Tpd52l1*) [53]. Hence, a role for OPP in preventing or slowing down ageing of the heart is implicated.

It was also noted that genes up-regulated in the hearts of ageing B6C3F_1 _mice such as aminolevulinic acid synthase 1 (*Alas1*), mature T cell proliferation 1 (*Mtcp1*) and protein tyrosine phosphatase 4a2 (*Ptp4a2*) [53], were down-regulated by OPP in the heart in the present study. In the group that had caloric restriction on the other hand, cyclin D2 (*Ccnd2*), secreted acidic cysteine rich glycoprotein (*Sparc*) and transferrin receptor (*Tfrc*) genes were down-regulated [53]. In the present study, these three genes were also down-regulated by OPP. It has been found that caloric restriction confers rapid positive effects on the heart, by rapidly shifting gene expression towards a state of reduced cardiac remodelling and fibrosis as well as enhanced contractility [54]. Several plant phenolics such as resveratrol, quercetin, butein and piceatannol have been proposed as potential caloric restriction mimetics [1]. The regulation of genes by OPP in the heart suggests that they may also act as caloric restriction mimetics.

In addition to genes similarly affected by caloric restriction, antioxidant genes such as those encoding erythroid derived-nuclear factor 2-like 1 (*Nfe2l1*), glutamate-cysteine ligase (*Gclm*) and various glutathione S-transferases (*Gstm2, Gstm5, Gstm 6*) were up-regulated. Glutathione S-transferases are important antioxidant enzymes essential in the detoxification of carcinogens and scavenging of ROS [40,55,56]. The up-regulation of these antioxidant genes thus indicates that OPP provides a higher antioxidant defence in the heart, which is an organ prone to damage by prooxidants.

The expression levels of genes in the heart TCA cycle, such as those encoding proteins which produce NADH (*Mdh1*) and FADH_2 _(*Shdb*), were also down-regulated. *Suclg1 *and *Sucla2*, genes encoding subunits of succinate-CoA ligases which convert GTP/ATP to GDP/ADP were also down-regulated. Finally, genes in the electron transport chain which encode proteins in Complex I, Complex II and Complex V were down-regulated as well. Electrons derived from metabolic reducing equivalents (NADH and FADH_2_) are fed into the electron transport chain through either Complex I or Complex II, and eventually pass to molecular oxygen to form water in Complex IV, while ATP is being formed in Complex V. These results imply that energy production in the heart was down-regulated by OPP. Hence, in reducing the amount of energy generated, OPP may act as antioxidants in reducing the amount of ROS produced in the heart, suggesting a beneficial effect of the extract in preventing cardiac oxidative stress.

### Summary and implications of the study

The discovery of OPP opens up a niche for value-added novel nutraceuticals. In this study, we confirmed that OPP was not toxic to mice as these compounds did not significantly alter the organ histology of the animals as well as their haematology and clinical biochemistry parameters. In addition, this study identified the global gene expression profiles caused by OPP in three major organs of mice on a normal diet. Supplementation of OPP to these mice resulted in numerous beneficial biological activities during healthy states. In livers of these mice, lipid catabolism genes were up-regulated while cholesterol biosynthesis genes were down-regulated, suggesting that OPP may find potential applications as hepatoprotective and anti-dyslipidaemic supplements. OPP may also act as possible anti-thrombotic agents since they encouraged the aggregation of platelets in spleens. OPP also retarded the ageing process in hearts, similar to the effects of caloric restriction.

Although the clinical biochemistry parameters on lipid profiles (triglycerides, total cholesterol, low-density lipoproteins and high-density lipoproteins) measured in this study did not show any significant differences between the groups, mice in the treatment group did show a delay in weight gain throughout the six weeks of feeding compared to the control group. Also, while the blood serum lipid profiles measured in the present study did not show any significant differences between the control and treated mice even though the hepatic gene expression profiles had changed, a similar observation was made by Matsui *et al*. (2005) when cocoa was given to mice [57]. In this previous study, cocoa ingestion increased total cholesterol concentrations in the blood sera, whereas body weight gain and hepatic cholesterol biosynthesis were decreased [57]. Hence, the difference between the clinical biochemistry parameters and the hepatic gene expression changes observed in the present study might be caused by other factors, which include reabsorption of cholesterol in the small intestine or other roles that the small intestine may play in the aetiology of obesity [58]. Perhaps the lipid profiles of livers in addition to blood sera should be conducted in future studies in order to directly correlate these profiles with the hepatic gene expression changes observed.

To our knowledge, this is the first study in which microarray analysis has been carried out on major organs of mice supplemented with OPP. In identifying possible pharmacological, nutraceutical or toxicological responses, the biological representation of a set of genes is more interesting than the genes themselves. By using different microarray softwares for statistical and functional analyses, we identified genes, gene ontologies, pathways and networks that were significantly changed by OPP in different major organs of mice on a normal diet. The gene expression profiles obtained indicate several mechanisms as to how OPP exerts its beneficial effects *in vivo*. These findings implicate broader pleiotropic effects of OPP than previously expected. This study also serves as a model for the dissection of many other complex responses mediated by dietary phenolics and plant phytochemicals in general.

## Conclusions

Our study was designed to understand the global functional profiles of OPP in mice which were on a normal diet. This analysis shows some of the molecular mechanisms as to how OPP exerts biological activities *in vivo *during healthy states. It thus provides a background for further hypotheses to be tested in future experiments involving OPP specifically, and dietary phenolics in general. This study also sets the scene for identifying the molecular modes of action involved in conferring the positive biological effects of OPP during various diseased states.

## Methods

### OPP samples

The OPP samples used in this study were prepared according to the methods described in Sambanthamurthi *et al*. (2008) [19]. OPP contains numerous phenolic acids, with three isomers of caffeoylshikimic acid as major components [20,21]. Other phenolic acids present include protocatechuic acid and *p*-hydroxybenzoic acid. The detailed composition of OPP is as described earlier [21].

### Animals, diets and treatments

All male inbred BALB/c mice which were designated for this study (*n = 20*) were purchased from the Institute of Medical Research, Kuala Lumpur, Malaysia, at around five weeks of age just after weaning. All animal procedures were approved by the Animal Care and Use Committee of the University of Malaya, Kuala Lumpur, Malaysia. The animals were randomly assigned into cages (*n = 5 *per cage) and acclimatised for one week, during which a standard chow diet purchased from the University of Malaya and distilled water, were given. At the start of the experiment, the diet of the animals was changed to a custom-made normal diet (58.2% kcal/kcal carbohydrate, 27.2% kcal/kcal protein and 14.6% kcal/kcal fat, including cellulose, mineral mix, vitamin mix and DL-methionine).

The control group (*n = 10*) was supplemented with distilled water while the treatment group (*n = 10*) was supplemented with OPP, as drinking fluids *ad libitum*. The antioxidant content of the OPP given was 1500 ppm gallic acid equivalent. Food and fluids were changed daily. During the animal feeding process, body weights were monitored every week, while fluid intake was monitored every day, for six weeks. Food intake and faecal output were monitored for seven consecutive days between week two to week three.

After six weeks, the mice were sacrificed via euthanasia with diethyl ether followed by exsanguination. Blood samples were collected after an overnight food fast (with fluids still provided) via cardiac puncture, while three major organs including livers, spleens and hearts were excised, rinsed in 0.9% w/v sodium chloride solution and weighed. Half of the organs (*n = 5*) were preserved in 10% formalin diluted with phosphate buffered saline for histology analysis, while the other half (*n = 5*) were snap-frozen in liquid nitrogen and stored at -80°C until the total RNA extraction process for gene expression analysis.

### Haematology analysis on blood samples

About 200 μL from half of each blood sample collected (*n = 4*) was aliquoted into a blood collection tube containing ethylenediaminetetraacetic acid to prevent clotting. These whole blood samples were sent immediately after dissection of the animals to the Clinical Biochemistry and Haematology Laboratory, Department of Veterinary Pathology and Microbiology, Faculty of Veterinary Medicine, University of Putra Malaysia, Serdang, Selangor, Malaysia, for haematology analysis. The analysis was carried out using the Animal Blood Counter Vet Haematology Analyser (Horiba ABX, France).

### Clinical biochemistry analysis on blood samples

In order to obtain sera, blood samples were allowed to clot at room temperature for two hours before being centrifuged at 1000 *xg *for five minutes, after which the supernatant layers were collected and stored at -20°C. The blood serum samples obtained were then sent for clinical biochemistry analysis using the Roche/Hitachi 902 Chemistry Analyser (Roche/Hitachi, Switzerland) in the Clinical Biochemistry and Haematology Laboratory, Department of Veterinary Pathology and Microbiology, Faculty of Veterinary Medicine, University of Putra Malaysia. Clinical biochemistry parameters which were examined include alanine aminotransferase, aspartate aminotransferase, glucose, serum total protein, albumin, globulin, ratio of albumin to globulin, total cholesterol, triglycerides, low-density lipoproteins and high-density lipoproteins. Of a total of ten serum samples per group obtained for clinical biochemistry analysis, two samples in the control group and three samples in the treatment group were excluded due to blood lysis.

### Histology analysis on organs

Histology analysis on dissected organs (*n = 5*) preserved in 10% buffered formalin was carried out in the Department of Pathology, Faculty of Medicine, University of Malaya, using standard routine procedures with haematoxylin and eosin staining.

### Total RNA extraction for gene expression analysis

All precautions in handling RNA were taken in this study. Total RNA isolation from mouse organs (*n = 5*) was carried out using the RNeasy Mini Kit (Qiagen, Inc., Valencia, CA) and QIAshredder homogenizers (Qiagen, Inc., Valencia, CA), preceded by grinding in liquid nitrogen using mortars and pestles. The total RNA samples obtained were subjected to NanoDrop 1000A Spectrophotometer (Thermo Fisher Scientific, Waltham, MA) measurement for yield and purity assessment. Integrity of the total RNA samples was assessed using the Agilent 2100 Bioanalyzer (Agilent Technologies, Santa Clara, CA) and Agilent RNA 6000 Nano Chip Assay Kit (Agilent Technologies, Santa Clara, CA). Four total RNA samples with the highest RNA Integrity Numbers and 28S/18S rRNA ratios within each condition (either control or treatment) were then selected for microarray studies.

### Microarray hybridisation, washing and scanning

Amplification of total RNA samples which were of high yield, purity and integrity was carried out using the Illumina TotalPrep RNA Amplification Kit (Ambion, Inc., Austin, TX). The cRNA produced was then hybridised to the Illumina MouseRef-8 Version 1 Expression BeadChip (Illumina, Inc., San Diego, CA), using the Direct Hybridization Kit (Illumina, Inc., San Diego, CA). Microarray hybridisation, washing and scanning were carried out according to the manufacturer's instructions. The raw gene expression data obtained are available at Gene Expression Omnibus [59] (Accession number: GSE28824).

### Microarray data analysis

Quality control of the hybridisation, microarray data extraction and initial analysis were carried out using the Illumina BeadStudio software (Illumina, Inc., San Diego, CA). Outlier samples were removed via hierarchical clustering analysis provided by the Illumina BeadStudio software and also using the TIGR MeV software (Institute for Genomic Research, Rockville, MD) [60], via different distance metrics. A minimum of three biological replicates per condition (with outliers removed) was then considered for further analysis. For livers, four replicates in the control group and three replicates in the treatment group were analysed. For spleens and hearts respectively, four replicates in the control group and four replicates in the treatment group were analysed.

Gene expression values were normalised using the rank invariant method and genes which had Detection Levels of more than 0.99 in either the control or treatment samples were considered significantly detected. To filter the data for genes which changed significantly in terms of statistics, the Illumina Custom error model was used and genes were considered significantly changed at a |Differential Score| of more than 20, which was equivalent to a P Value of less than 0.01 [61].

The genes and their corresponding data were then exported into the Microsoft Excel software (Microsoft Corporation, Richmond, WA) for further analysis. To calculate fold changes, an arbitrary value of 10 was given to expression values which were less than 10. Fold changes were then calculated by dividing the mean values of Signal Y (treatment) with those of Signal × (control) if the genes were up-regulated and *vice versa *if the genes were down-regulated. Two-way (gene and sample) hierarchical clustering of the significant genes was then performed using the TIGR MeV software to ensure that the replicates of each condition were clustered to each other. The Euclidean distance metric and average linkage method were used to carry out the hierarchical clustering analysis.

Changes in biological pathways and gene ontologies were assessed via functional enrichment analysis, using the GenMAPP [24] and MAPPFinder [25] softwares (University of California at San Francisco, San Francisco, CA). The MAPPFinder software ranks GenMAPPs (pathways) and gene ontologies based on the hypergeometric distribution. Readers are referred to Doniger *et al*. (2003) [25] for further explanations of the terms used in the MAPPFinder software. GenMAPPs and gene ontologies which had Permuted P Values of less than 0.01, Numbers of Genes Changed of more than or equal to 2 and Z Scores of more than 2 were considered significant.

It should be noted that the MAPPFinder software clusters multiple probes for a distinct gene into a single gene grouping in order to calculate the number of distinct genes which meet the user-defined criteria, not the probes. In this study, up-regulated and down-regulated genes were analysed separately in the functional enrichment analysis but were viewed together in each GenMAPP. Boxes coloured yellow indicate genes which were up-regulated while those coloured blue indicate genes which were down-regulated. The fold changes are indicated next to the boxes. Individual boxes which have different shadings within them indicate the presence of multiple probes (splice transcripts) within a single gene.

Changes in regulatory networks were also analysed through the use of the Ingenuity Pathways Analysis software (Ingenuity^® ^Systems, Redwood City, CA) [26]. For each organ, a dataset containing differentially expressed genes and their corresponding fold changes was uploaded into the application. Analyses of up-regulated and down-regulated genes were carried out separately. Each gene identifier was mapped to its corresponding gene object in the Ingenuity Pathways Knowledge Base. These genes were overlaid onto a global molecular network developed from information contained in the Ingenuity Pathways Knowledge Base. Networks of these focus genes were then algorithmically generated based on their connectivity.

A network is a graphical representation of the molecular relationships between genes or gene products. Genes or gene products were represented as nodes, and the biological relationship between two nodes was represented as an edge (line). The intensity of the node colour indicates the degree of up-regulation (red) or down-regulation (green). Nodes were displayed using various shapes that represented the functional class of the gene product. Edges were displayed with various labels that described the nature of the relationship between the nodes.

### Real-time qRT-PCR validation

Two-step real-time qRT-PCR studies were carried out on six target genes selected to represent the different organs used in the microarray experiments, using TaqMan Gene Expression Assays (Applied Biosystems, Foster City, CA). These genes were selected based on their presence in significant functions, their differential scores, their detection levels, their presence as single splice transcripts in microarrays and their availability as Taqman assays designed across splice junctions. The same aliquots of total RNA samples used in the microarray experiments were utilised for this analysis. Primer and probe sets for the selected genes were obtained from the Applied Biosystems Inventoried Assays-On-Demand (Applied Biosystems, Foster City, CA).

Briefly, reverse transcription to generate first-strand cDNA from total RNA was carried out using the High-Capacity cDNA Reverse Transcription Kit (Applied Biosystems, Foster City, CA). Real-time PCR was then carried out on the first-strand cDNA generated using a 25 μL reaction volume in an Applied Biosystems 7000 Real-Time PCR System (Applied Biosystems, Foster City, CA), with the following conditions: 50°C, 2 minutes, 1 cycle; 95°C, 10 minutes, 1 cycle; 95°C, 15 seconds and 60°C, 1 minute, 40 cycles. For gene expression experiments, reactions for each biological replicate and non-template control (NTC) were carried out in duplicates. For amplification efficiency determination, reactions were carried out in triplicates.

Quality control of the replicates used, real-time qRT-PCR data extraction and initial analysis were carried out using the 7000 Sequence Detection System software (Applied Biosystems, Foster City, CA). A manual threshold of 0.6000 and an auto baseline were applied in order to obtain the threshold cycle (Ct) for each measurement taken. The threshold was chosen as it intersected the exponential phase of the amplification plots [62]. The criteria for quality control of the data obtained include ΔCt of less than 0.5 between technical replicates and ΔCt of more than 5.0 between samples and NTCs [63].

Relative quantification of the target genes of interest was carried out using the qBase 1.3.5 software (Center for Medical Genetics, Ghent University Hospital, Ghent, Belgium) [64], which takes into account the calculations of amplification efficiencies and multiple housekeeping genes. Expression levels of target genes were normalised to the geometric mean of three housekeeping genes, arginine/serine rich splicing factor 9 (*Sfrs9*), guanylate kinase 1 (*Guk1*) and heterogeneous nuclear ribonucleoprotein A/B (*Hnrpab*). These genes were chosen as they were shown to be stable across the previously obtained microarray data, and confirmed to be more stable than 18S ribosomal RNA (*18SrRNA*), via the usage of the geNorm 3.5 software (Center for Medical Genetics, Ghent University Hospital, Ghent, Belgium) [65].

### Statistical analysis

Statistical analysis was carried out by using the two-tailed unpaired Student's t-test available in the Microsoft Excel software (Microsoft Corporation, Redmond, WA) unless otherwise stated. Differences with P Values of less than 0.05 were considered statistically significant.

## List of abbreviations

ADP: adenosine diphosphate; ATP: adenosine triphosphate; CCAAT: cytidine-cytidine-adenosine-adenosine-thymidine; CoA: coenzyme A; cDNA: complementary deoxyribonucleic acid; cRNA: complementary ribonucleic acid; Ct: threshold cycle; DNA: deoxyribonucleic acid; FADH_2_: flavin adenine dinucleotide (fully reduced or hydroquinone form); Fe: iron; GDP: guanosine diphosphate; GenMAPP: Gene Map Annotator and Pathway Profiler; GTP: guanosine triphosphate; HMG: 3-hydroxy-3-methyl-glutaryl; ID: identifier; MAPP: Map Annotator and Pathway Profiler; MAPPFinder: Map Annotator and Pathway Profiler Finder; MeV: MultiExperiment Viewer; mRNA: messenger ribonucleic acid; NAD(P): nicotinamide adenine dinucleotide phosphate; NADH: nicotinamide adenine dinucleotide (reduced form); NDH: Normal Diet - Hearts; NDL: Normal Diet - Livers; NTC: non-template control; O: oxygen; OPP: oil palm phenolics; *p: para; *P: phosphate; PP: pyrophosphate; qRT-PCR: quantitative reverse transcription-polymerase chain reaction; RNA: ribonucleic acid; ROS: reactive oxygen species; rRNA: ribosomal ribonucleic acid; S: sulphur; s.e.m.: standard error of the mean; TCA: tricarboxylic acid; TIGR MeV: The Institute for Genomic Research; UDP: uridine diphosphate

## Authors' contributions

SSL carried out the animal feeding, collected the samples, carried out the histology, microarray and real-time qRT-PCR experiments, performed the data analysis, interpreted the data and drafted the manuscript. SDS helped in the interpretation of the microarray data, in addition to supervising the study. KS designed the animal feeding. YAT was involved in the preparation of OPP. RS conceived the study and participated in its design and coordination, in addition to supervising the study. All authors participated in helpful discussions and read as well as approved the final manuscript.

## Supplementary Material

Additional file 1 **List of genes significantly changed in the liver, spleen and heart by OPP**. The |Differential Score| for all genes is more than 20, equivalent to a P Value of less than 0.01. Click here for file

Additional file 2 **An example of the two-way hierarchical clustering analysis carried out on microarray data (using data from the liver as an example)**. In this figure, single colour gene expression values are represented using a blue-white-yellow (0 to positive) colour scheme. Grey boxes indicate negative values. Note that the replicates of each group (LA1, LA2, LA4, LA5 in the control group and LB1, LB2, LB4 in the treatment group) were clustered together within each group but separated from the other group, indicating that the gene expression values selected as significantly changed differentiated the two groups. Click here for file

Additional file 3 **List of GenMAPPs and gene ontologies significantly changed in the liver, spleen and heart by OPP**. All GenMAPPs and gene ontologies had Permuted P Values of less than 0.01, Numbers of Genes Changed of more than or equal to 2 and Z Scores of more than 2. Click here for file
