# Supplementary figures and images for: Differential transcriptomic profiles effected by oil palm phenolics indicate novel health outcomes
Source: BMC Genomics. 2011 Aug 25;12:432. doi: 10.1186/1471-2164-12-432 (PMC3175228; doi:10.1186/1471-2164-12-432)

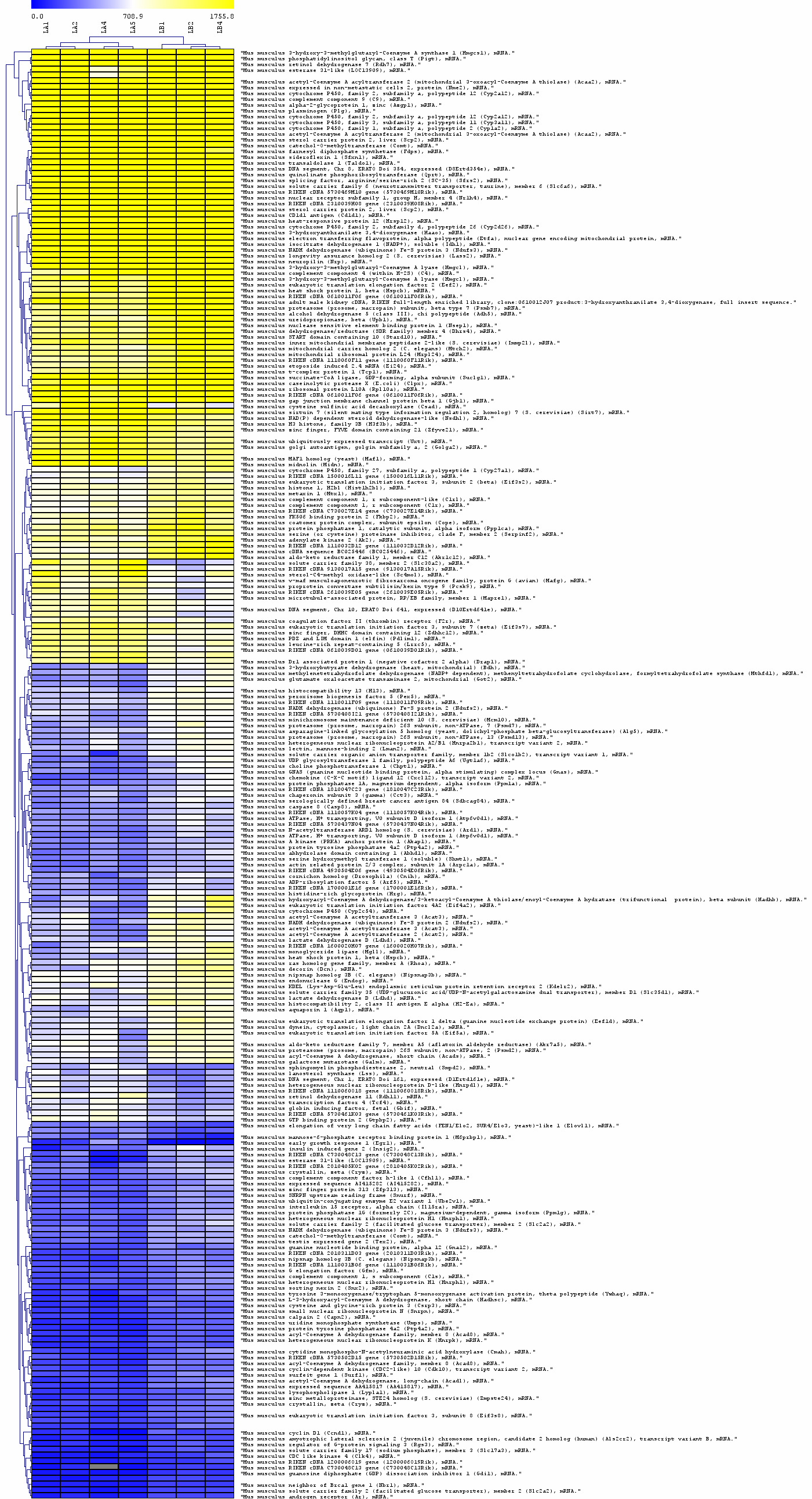

Supplement: Additional file 2 — An example of the two-way hierarchical clustering analysis carried out on microarray data (using data from the liver as an example). In this figure, single colour gene expression values are represented using a blue-white-yellow (0 to positive) colour scheme. Grey boxes indicate negative values. Note that the replicates of each group (LA1, LA2, LA4, LA5 in the control group and LB1, LB2, LB4 in the treatment group) were clustered together within each group but separated from the other group, indicating that the gene expression values selected as significantly changed differentiated the two groups. [file 1471-2164-12-432-S2.PNG]
